# Assessment of Concentrations of Heavy Metals and Phthalates in Two Urban Rivers of the Northeast of Puerto Rico

**DOI:** 10.4172/2161-0525.1000353

**Published:** 2016-03-20

**Authors:** Ana I Ortiz-Colón, Luis E Piñero-Santiago, Nilsa M Rivera, María A Sosa

**Affiliations:** 1 Department of Anatomy and Neurobiology, School of Medicine, Medical Science Campus, University of Puerto Rico, San Juan, Puerto Rico, 00936 USA; 2 Institute of Neurobiology, Medical Sciences Campus, University of Puerto Rico, San Juan, Puerto Rico, 00901 USA; 3 Puerto Rico Center for Environmental Neuroscience, Medical Sciences Campus, San Juan, Puerto Rico, 00936 USA; 4 Department of Chemistry, Humacao Campus, University of Puerto Rico, Humacao, Puerto Rico, 00792 USA

**Keywords:** Urbanism, Emergent contaminants, Water pollution, Plasticizers, Consumer products solvents

## Abstract

Urbanization adjacent to rivers has increased in recent years and is considered a source of environmental contamination. The resulting increase in number of urban rivers in highly populated areas, such as the Caribbean island of Puerto Rico, has led to the appearance of synthetic as well as naturally occurring chemicals not previously observed nor regularly monitored in freshwater habitats. Some of these chemicals, such as heavy metals and plasticizers, have been shown to affect endocrine, respiratory, and nervous system function in animals and humans, even at relatively low concentrations. The purpose of this study was to measure concentrations of such emergent contaminants on rivers of urbanized areas on the northeast of Puerto Rico, as one element in the assessment of the impact of urbanism on water quality in these communities. To accomplish this, we used Inductively Coupled Plasma and Gas Chromatography Mass Spectrometry to measure amounts of heavy metals and phthalates, respectively, in superficial water of three rivers of Puerto Rico: Mameyes (non-urban), Río Piedras (urban river without a dam), and La Plata (urban river with a dam). The urban rivers had significantly higher concentrations of heavy metals arsenic, barium, cadmium, manganese, and antimony, when compared with the reference non-urban river. Manganese was the only metal found in concentrations higher than limits established by the EPA for drinking water. Of eight phthalates amenable to measurement with the chosen protocol and instrumentation, only dibutyl phthalate was detected, only in the La Plata river, and at concentrations ranging from 3 to 8 parts-per-billion. These findings suggest that urbanism close to rivers of Puerto Rico is likely having an impact on water quality and thus further study to identify the potential sources, as well as the inclusion of these emergent contaminants on the list of chemicals regularly monitored by government agencies is justified.

## Introduction

The continuous increase of the population and social, economic, and infrastructure development experienced in Latin American countries and the Caribbean in recent decades has triggered a rapid urban growth, where many inhabitants have migrated from rural areas to concentrate in urban zones. The urbanization process in these countries has reached a level where their urban region is considered as one of the most urbanized worldwide, along with North America and Europe. In 1950, the urban population in Latin America and the Caribbean was 41.4%, increasing by 1995 to 73.4%, and around 79% by 2010. It is estimated that by the year 2030 the urban population in these countries will be 83.2% [1]. In the next 30 years, a similar pattern of increased urbanism is expected in other continents like Asia and Africa [2].

Puerto Rico, a self-governing dependent territory of the United States (US) with an area of 8,870 km^2^, is considered one of the oldest urban regions in the tropics. During the decade of the 1940s, the Puerto Rican economy was based mostly on agriculture. Throughout the 1960s the island’s economy initiated a transition from agriculture towards industrialization, a period during which people started moving from rural to urban areas. In 2014 the island’s population was estimated at 3.548 million inhabitants, with an average density of 400 inhabitants/km^2^, among the world’s highest. This corresponds to position #24, according to the World Bank [3], amongst a list of the 214 sovereign states and self-governing dependent territories of the world today, and position #3, according to the US Census, within the states and territories of the United States [4].

The urban growth of Puerto Rico and Latin America has been characterized by its ample expansion around metropolitan centers. In Puerto Rico, urban growth is more evident near the metropolitan area of the municipalities of San Juan-Bayamón-Carolina [5], while in Latin America it is more prevalent in the cities of Panamá and Guatemala, San José in Costa Rica, Santiago in Chile, Lima in Perú, and Buenos Aires in Argentina [6].

The urbanistic process involves the progressive conversion of natural ecological systems to urban ones [7]. This process often is considered a threat to the surrounding ecosystems [8] because of the alteration of the atmospheric composition, impact on the hydrology of rivers, the geomorphology of beds of water bodies, and accumulation of contaminants in the ground cover, eventually also reaching water bodies. In addition, urbanism is associated with various sources of contamination such as industrial waste, fluids from heating and refrigeration systems, transport systems, water waste, collection and disposal of solids, industrial plant waste and sewer runoff, among others.

The impact of urbanism on rivers is due primarily to changes on the routes of their tributaries and channels and its bank walls to make them fit to the layout of housing and businesses. These changes increase land impermeable areas that prevent normal ground percolation of rain water, and result in fast washing towards the rivers of pollutants generated by humans living in these urban areas, including solvents, dyes, household products, fluids and particulates from automobiles, fertilizers and pesticides used in parks, gardens and green areas, etc. [7]. Studies have been carried out in various countries regarding contamination of rivers as a result of urbanism and associated anthropogenic activities.

Traces of manganese (Mn), iron (Fe), cobalt (Co), bromine (Br), copper (Cu), zinc (Zn), lead (Pb), cadmium (Cd), chromium (Cr), nickel (Ni), and arsenic (As) have been identified in various freshwater bodies throughout the world [1,9–31]. In Canada and the states of Washington and Oregon of the US, the Columbia River, the largest river in the Pacific Northwest region of North America, has been contaminated with heavy metals Cu, Cd, and Zn from metallurgic industries, discharge from municipalities, and mines [1]. Similar urban contamination with metals has been reported in the Marcal River of Hungary, rivers of Southeast Asia, west Finland, southeast Brazil, Serbia and central Nigeria, as well as in tributaries of the Nile River in Egypt. The metals most commonly found in these rivers have been Cr, Zn, Cd and Cu [9–17]. Heavy metals in water pose a worrisome health threat because they tend to be taken up by fish [10,29,32–38] and other animals that are used as food sources for humans, such as mollusks and crustaceans [19,39–46].

The Yangtze river of China, the third longest of the world, is considered an urban river because the activity of 8 million inhabitants is concentrated on its river banks. In the past 10 years it has been characterized by the presence of high concentrations of polychlorinated biphenyls (PCBs) [23], organic contaminants coming from plastics. PCBs have been shown to affect several biological processes such as chondrocyte death, cell death involving mechanisms of apoptosis, necrosis and oxidative stress, and have also been associated with impairment of intellectual ability during early human development and neurobehavioral alterations in newborns [47–52].

A recent (2014) review article by Cousins and colleagues [53] summarizes evidence compiled between 1973 and 2008 on the presence of various phthalates, other components of plastics, including diethyl phthalate (DEP), dibutyl phthalate (DBuP), di-(2-ethylhexyl) phthalate (DEHP), and benzylbutyl phthalate (BzBuP), in outdoor and indoor air in different countries in North America, Europe and Asia. The origin of the majority of phthalates detected in these studies was found in domestic dust, an important source in highly urbanized areas that can easily contaminate air and water in close-by environments. Other studies looking at organic contaminants in bodies of water have identified phthalates as well. For example, a 2011 study of the Yangtze River, the main source of drinking water for the inhabitants of China and other Asian countries, found organic pesticides, aromatic hydrocarbons, polychlorinated biphenyls and phthalates, including DEP, DBuP, and DEHP [54], all linked to industries related with the manufacture of automobiles, fabrics, and electronics. Although thus far the concentrations of these contaminants in the Yangtze River are below the amounts allowed by government standards for surface water, concerns remain because the effects of prolonged exposure to low levels of these chemicals is not well characterized yet.

Research on the impact of urbanism and contamination in other parts of the world has shown that this phenomenon causes degradation of river ecosystems. However, studies of this type in tropical regions have been poorly documented [8,55]. Puerto Rico, as a small tropical island with urban development booming since the 1950s [5] is an ideal location to assess the impact of urbanism and contamination in various habitats or environments, including rivers, because a wide variety of ecosystems are found within relatively short distances. The resources from freshwater ecosystems directly contribute to the survival of the Puerto Rican population and attract many people to settle on or near river banks. However, contamination of rivers due to increase in urbanization can negatively impact the health of humans and other animal species. This study seeks to measure amounts of metal and phthalates in three Puerto Rico Rivers surrounded by varying levels of urbanism to aid in evaluating how urbanism close to freshwater systems can affect water quality [56,57].

## Materials and Methods

### Sample collection

Surface water samples were collected from two sampling sites at a non-urban or reference river, the Mameyes, and from three sampling sites in each of two urban rivers, the Río Piedras and Río La Plata, between May and November of 2013, which corresponds with the rainy season in Puerto Rico.

Water samples from each of the river’s sampling points were taken in triplicate, as described in S.E Long (Technology Applications INC.), and in the Environmental Protection Agency’s Methods 200.8 [58] and 200.2 [59], with few modifications. Clean one-liter amber plastic or glass bottles were used to collect water samples for analysis of metals and phthalates, respectively. Control sample bottles of each kind, containing ultra-pure water from the laboratory, were also taken to each sampling point, where they were handled in the same way as the river water sample bottles (including storage, transportation, and opening of the bottle on location, with the only difference that no river water is added).

Physicochemical properties of the water, such as temperature and pH, were measured at each sampling point using an YSI Water Quality Meter (Model 63, YSI Incorporated, Yellow Springs, OH), while dissolved oxygen at each site was measured using YSI’s ProODO Optical Digital Handheld Instrument.

### Processing and analysis of metals in the river water samples

Amounts of various metals of interest, specifically silver (Ag), arsenic (As), barium (Ba), beryllium (Be), cadmium (Cd), chromium (Cr), manganese (Mg), nickel (Ni), lead (Pb), antimony (Sb), selenium (Se), thallium (Tl) and zinc (Zn), were analyzed in the surface water samples using Inductively Coupled Plasma – Mass Spectrometry (ELA 6100 model, Perkin Elmers, Waltham, MA) as described in US EPA Method 200.8 [59], with a few modifications in accordance with the instrument’s specifications.

Briefly, volumes of 50 ml of each water sample were treated with 1 ml of nitric acid (HNO_3_ 70%, purified by re-distillation, 99.999+ % trace metals basis 1+1, Chemical and Chromatography Supplies, Inc., San Juan, PR) and 0.5 ml of hydrochloric acid (HCl 35–38% 1+1, Fisher Scientific, Bridgewater, NJ), and digested and evaporated at 85°C for 4.5 hours. Volumes of 15–20 mL of each of these samples were then filtered to minimize the risk of any remaining solid residues from later on occluding the instrument. The filtration step is done after the digestion and evaporation process to make sure metals that may be adhered to solids in the sample are not lost during preparative processing. Distilled water was added to bring each sample to a final volume of 25 ml, they were mixed well, had 5 ml removed, followed by a final addition of 30 ml of distilled water, reaching a final sample volume of 50 ml (dilution factor of 1.25). Metal standards were obtained from ERA A Water Company (Colorado, USA).

### Processing and analysis of phthalates in the river water samples

Preservation of the river water samples to be analyzed for phthalate content was secured by adding 3 ml of nitric acid concentrate (0.1 M HNO_3_ in H_2_O [0.1N] eluent concentrate for ion chromatography [IC]; Chemical and Chromatography Supplies, Inc., San Juan, PR), followed by storage at room temperature. A solid phase extraction protocol was used prior to analysis, as described in Supelco Bulletin 910 (Sigma-Aldrich, St. Louis, MO) [57–61]. For this procedure a vacuum manifold (Sigma-Aldrich, St. Louis, MO) was used, fitted with Oasis Hydrophobic-Lipophilic Balance (HLB) glass cartridges (Part number: 186000116 Waters Technology, Caguas, PR), a vacuum pump, glass test tubes and glass Pasteur pipettes, using distilled water, ethyl acetate 99.999% (VWR Radnor, Pennsylvania, USA) and methanol 99.9% (ACS spectrophotometer grade, Sigma Aldrich, St. Louis, MO), stored in glass bottles.

Glass cartridges were conditioned with 10 mL of a 25% solution of methanol in distilled water, and then rinsed with 10 mL of ultra-pure water. Flow through the cartridge was adjusted to 1 mL/min to ensure the interaction of the particulate in the sample with the stationary phase. A volume of 300 mL of the sample was then gradually added, at this same flow rate. The solid phase of each cartridge was dried with a vacuum pump using nitrogen gas, and the components that bound to the stationary phase were extracted by adding 10 mL of ethyl acetate 99.999%. The resulting eluates were collected in labeled tubes, with sequential fractions being pre-concentrated with nitrogen gas until reaching a volume of 1 mL each.

Analysis of samples for detection of phthalates was performed as described by Li and colleagues [62–64] using a Gas Chromatograph (GC) 2000 (Thermo Finnigan, San Diego, CA) coupled to a Mass Spectrometric Detector (Polaris Q, Thermo Electron, Austin, TX). A DB5MS capillary column of 30 m, 0.25 mm X 0.320 mm was employed for analyte separation. Instrument conditions were: initial temperature of 100°C with a rate of change of 5 °C/minutes until reaching 250°C, with a total run time of 30 minutes. Mass spectra were obtained at an electron impact potential of 70 eV with a range of 30–500 amu.

Six phthalates, from a list of the eleven most commonly reported in the literature that were also commercially available, were chosen on the basis of the chemical properties that were most amenable to analysis using Gas Chromatography-Mass Spectrometry (GC-MS), namely volatility, thermal stability, and capacity for ionization (Table 1, chosen phthalates shown in *). Since it was thought likely that their concentrations in the river water samples could be near trace amounts, the minimum detection limit of the Polaris Q (MS) for phthalates was determined and found to be 0.01 mM. Initial screening or qualitative identification of the phthalates present in a river water sample was conducted using the instrument’s full scan mode, followed by characterization with the single ion monitoring (SIM) mode to quantify the desired compounds.

### Statistical analysis

Statistical comparisons amongst means of groups were performed by one-way ANOVA. When significant differences were detected through ANOVA, Tukey post hoc analysis was employed for individual comparisons. Results were presented as mean ± of 6–9 determinations from 2 to 3 experiments where p≤0.05 was considered significant. GraphPad Prism software version 6.0 (GraphPad Software, Inc., California, USA) was used for this statistical analysis.

## Results

### Sampling locations

**Figure 1:**
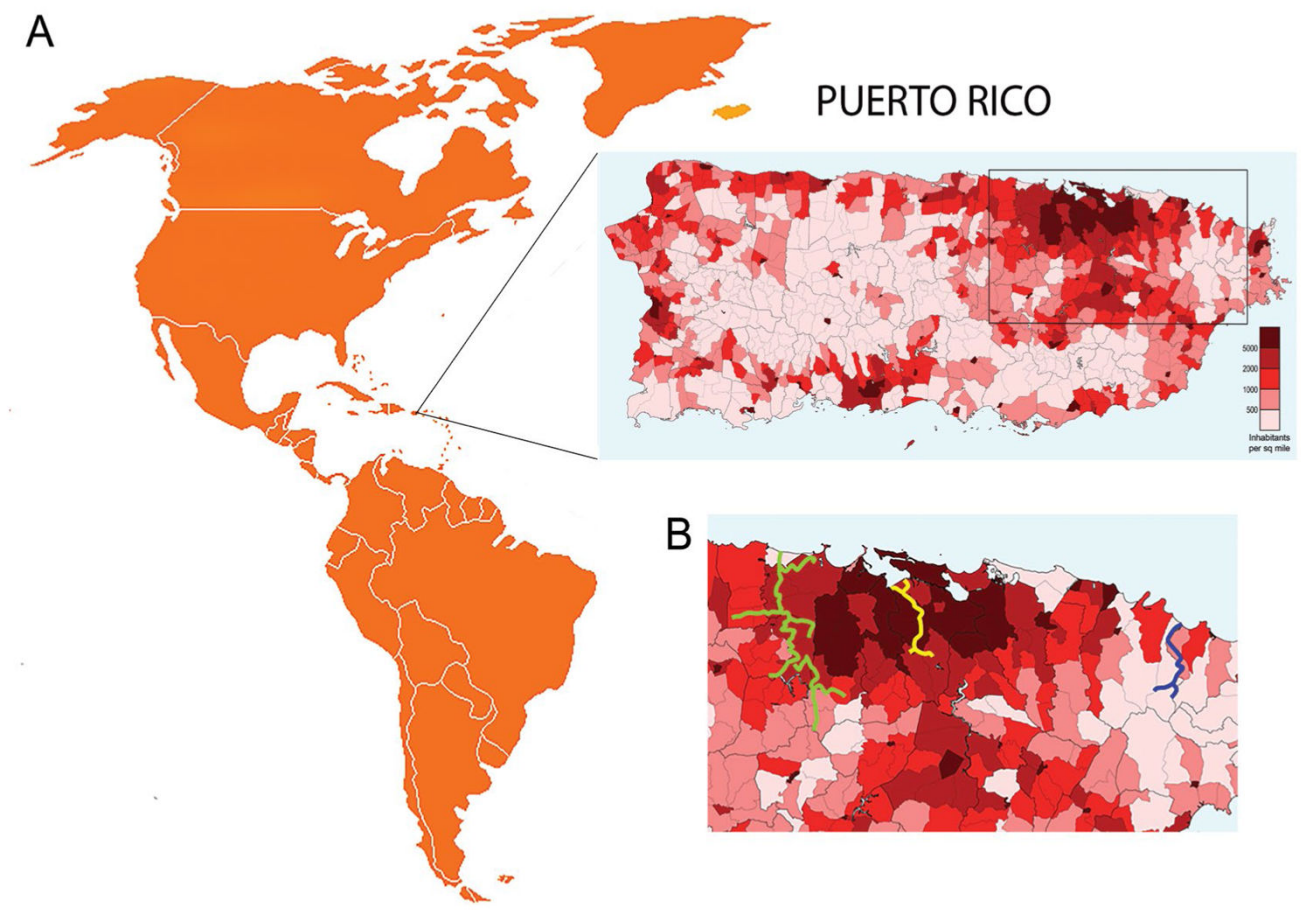
A. Map of Puerto Rico, showing location within the Greater Antilles archipelago. Enlarged map shows population density within regional subdivisions of municipalities. Color legend indicates number of inhabitants per square mile, based on the 2000 US Census data. The darker the color, the more densely populated the area is (map was created by Javier Rodríguez Galarza, copyright holder of the work under Creative Commons Attribution 3.0 License). B. Closer view of area shown in the rectangle on map A, showing location of selected rivers. Blue, yellow, and green colored lines represent the selected rivers: blue – Mameyes (non-urban); yellow – Río Piedras (urban without a dam); green – La Plata (urban with a dam).

Three rivers that flow towards the Northeastern coast of Puerto Rico were selected on the basis of the desired characteristics regarding urbanism, length and elevation. These rivers are the Mameyes, Río Piedras, and La Plata (Figure 1).

**Figure 2:**
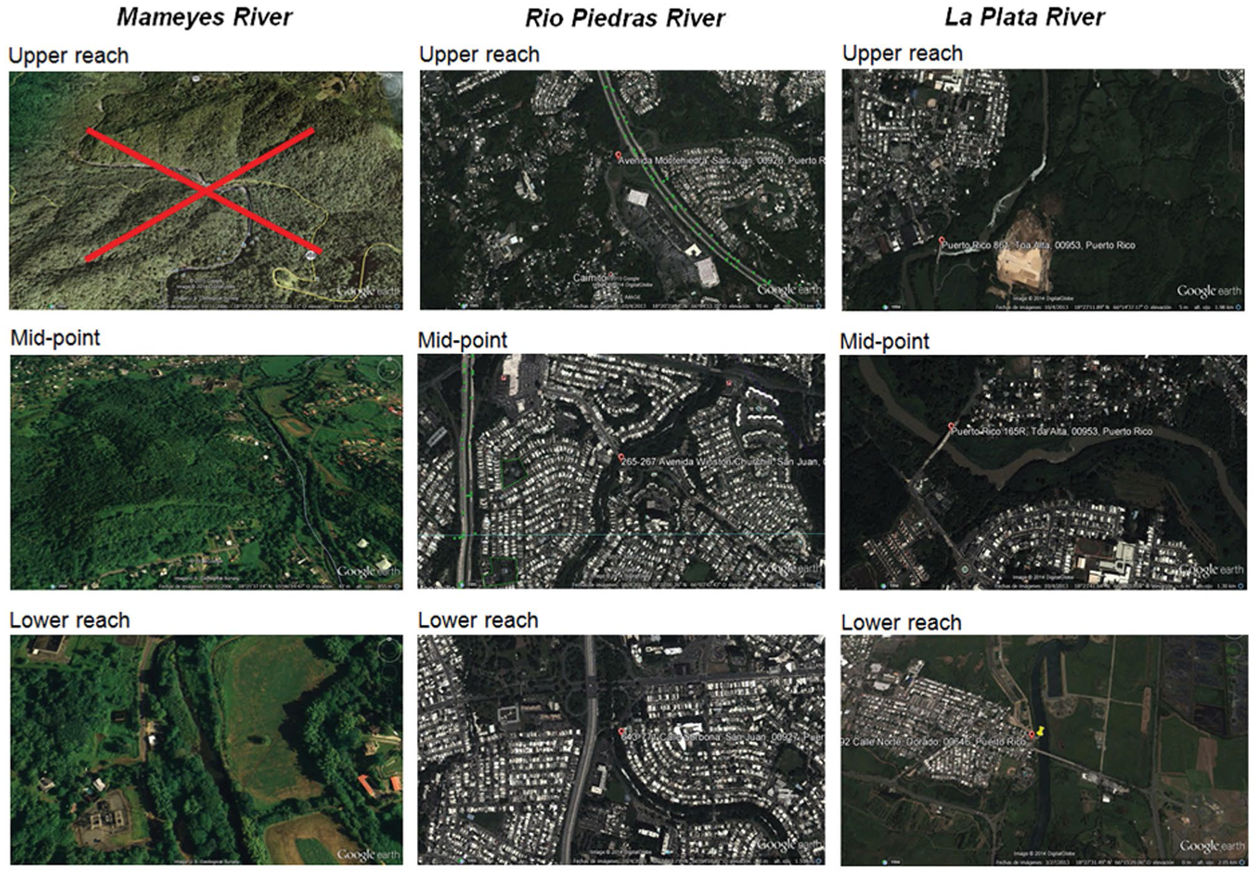
Satellite (Google Maps) images of the river sampling sites, illustrating the relative levels of urbanism at each. (Red X indicates that sampling was not possible at the upper reach of the Mameyes River because it corresponded to the interior to the Federal Reserve area).

The *Mameyes River,* selected as the non-urban reference site, is located in the municipality of Río Grande, Puerto Rico (Figure 2). The Mameyes has a continuous water flow, no dam and is within a rural area, with minimal anthropogenic influence because it is part of a biosphere reserve [56,57]. Sampling was conducted at its midpoint and a lower reach point near the river’s exit to sea, just before the estuarine waters. It was not possible to sample from the upper reach of this river because it is found within the US Federal Reserve area of the rainforest. The midpoint of the river is located in latitude 18°21′41.62″ North, longitude 65°46′7.58″ West, at an elevation of 12 meters and a depth of 23.3″. The access to this point was along Road 191 towards the El Yunque Rainforest, which runs parallel to the river. The chosen lower reach was at latitude 18°21′53.14″ North, longitude 65°46′11.42″ West, at an elevation of 10 meters and a depth of 15.7″. This point was farther from the road, near a water pumping station of the Aqueducts and Sewer Authority of Puerto Rico.

Two rivers were selected as examples of urban rivers, the Río Piedras and La Plata. The *Río Piedras River* is located in the capital city of San Juan, Puerto Rico (Figure 2). This river has a continuous stream and no dam. The selected sampling point near its upper reach was located at latitude 18°20′38.23′ North, longitude 66°4′13.18″ West, at an elevation of 69 meters and a depth of 16.3″, right next to a shopping mall. The river’s midpoint is at latitude 18°21′ 59.82′ North, longitude 66°03′48.25″ West, at an elevation of 30 meters and a depth of 7.7″. This sampling point was next to a very busy and crowded avenue, with various food establishments, clothing stores, schools, pharmacies, and residences nearby, with sewer runoffs from the area draining towards the river. The selected lower reach sampling point was at latitude 18°24′19.67″ North, longitude 66°04′08.46″ West, at an elevation of 3 meters and a depth of 10″. This point was near a major expressway and several exits to large avenues in the city’s metropolitan area.

The *La Plata River* is located in the municipalities of Dorado and Toa Alta, and has a regulated flow and a dam reservoir (Figure 2). The selected sampling point near its upper reach was located at latitude 18°22′59.29″ North, longitude 66°14′56.59″ West, at an elevation of 8 meters and a depth of 12″. Crossing over the river is a tall bridge with two eastbound and two westbound lanes. The river’s midpoint is at latitude 18°23′43.94″ North, longitude 66°15′15.44″ West, at an elevation of 4 meters and a depth of 58.3″. This sampling site was near a gas station, a garden center and some houses. The selected lower reach sampling point, located at the entrance to Dorado’s town and also crossed by a bridge that gives entry to the town’s center, was at latitude 18°27′29.86″ North, longitude 66°15′ 30.44″ West, at an elevation of 1 meter and a depth of 24.5″.

### Physicochemical parameters

Measures of the physicochemical parameters of pH, temperature, and dissolved oxygen at the three rivers at the time of sampling are shown in Table 2. These three parameters did not differ significantly along the length of each river. The pH values at the selected sampling points of the three rivers ranged from 7.39 at La Plata’s low reach to 8.16 at the Rio Piedra’s midpoint. The mean pH at Mameyes and La Plata were near neutral, while that at the Río Piedras was significantly more alkaline (*p=0.0007*). The temperatures at the three rivers, which ranged from 25.5°C at the Mameye’s midpoint to 31.3°C at the Río Piedras lower reach, did not differ significantly. The largest differences amongst the three rivers were observed in the parameter of dissolved oxygen, which ranged from 5.00 at La Plata’s lower reach to 9.15 mg/L at Río Piedras lower reach. The mean dissolved oxygen at La Plata was significantly lower than those of the Mameyes and the Río Piedras rivers.

### Heavy metals in superficial water samples

The mean concentration values at each collection point at the time of sampling of the group of fourteen heavy metals analyzed in the three rivers are shown in Table 3. Mean overall values (averages of collection sites) for each river, expressed as percentage of the maximum contaminant levels (MCL) established by the United States Environmental Protection Agency (EPA) for drinking water, are shown in Table 4.

Concentration ranges, classified as ≤ 1%, 1–10%, 10–25%, 25–100%, and >100% MCL, were similar amongst each collection point of each river, except in the case of Río La Plata, where concentrations for *As* were found at different % MCL ranges in one collection point.

At the Mameyes, the non-urban reference river, *Ag, Be, Cu, Se,* and *Zn* were found at or below 1% MCL, while *As, Ba, Cd, Cr, Ni*, *Pb, Tl* and *Sb* were in the range of 1–10% MCL, and *Mn* was found in the range of 10–25% MCL. At the Río Piedras, an urban river with no dams, the range of % MCL at which these metals were observed were similar to those found for the Mameyes, with the only difference that *Pb* was found at a lower range (below 1% MCL) and *Mn* was found at a higher range (25–100% MCL). At the upper and midpoint reaches of La Plata, an urban river with a dam, *Pb* was also found in a range different to that at Mameyes (below 1% MCL), while *Se* reached the 1–10% MCL range, and *As* reached that of 10–25% at the upper reach (as it also did at the lower reach). Manganese (*Mn*) at the three collection sites at La Plata was found at amounts that more than tripled or quadrupled the EPA’s MCL, these being the only sites where a heavy metal was observed to exceed the EPA’s maximum contaminant levels for drinking water.

**Figure 3:**
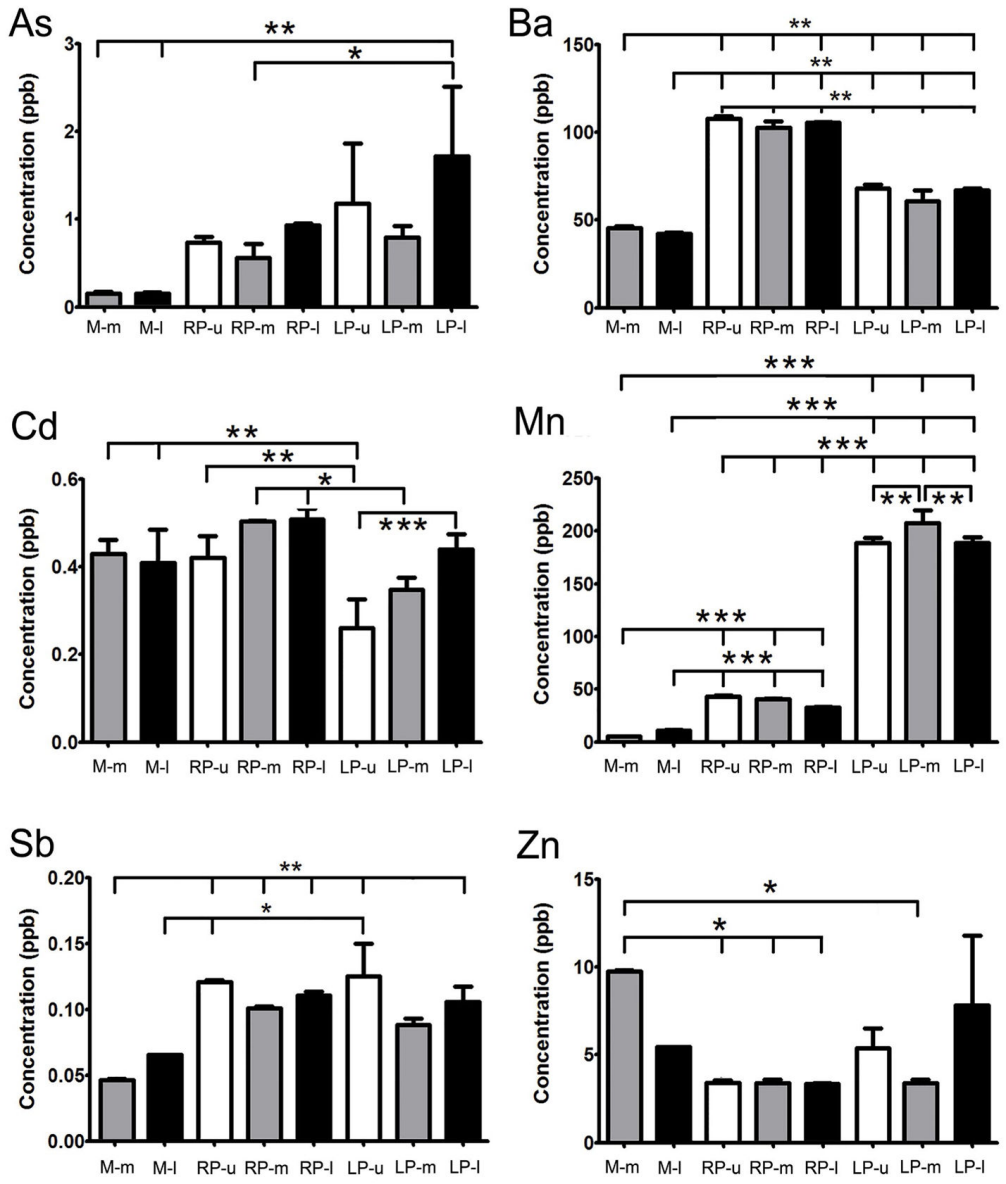
Comparison of heavy metal concentrations that were found to differ significantly amongst non-urban and urban rivers at the northeast of Puerto Rico during the 2013 rainy season. As=Arsenic; Ba=Barium; Cd=Cadmium; Mn=Manganese; Sb=Antimony; Zn=Zinc. Concentrations are shown in parts per billion. Brackets indicate comparisons where differences were significant (*p<0.05, **p<0.01, ***p<0.001). M=Mameyes river; RP=Río Piedras river; LP=La Plata river; u=upper reach; m=midpoint; l=lower reach.

Significant differences between the non-urban and urban rivers were observed in the concentrations of *As, Ba, Cd, Mn, Sb*, and *Zn* (Figure 3). The concentrations of *As, Ba, Mn*, and *Sb* were higher in both urban rivers, whereas the concentration of *Cd* was higher in the urban river without a dam but not the one with a dam, and the concentration of *Zn* was lower in both urban rivers. Significant differences between the two urban rivers, one with a dam (La Plata) and the other without (Río Piedras), were observed in the concentrations of *As, Ba, Cd* and *Mn* (Figure 3). The concentrations of *As* and *Mn* were higher at La Plata, the river with a dam, while those of *Ba* and *Cd* were higher at Río Piedras, the river without a dam. Significant differences between different collection sites within the same river were observed only for *Cd* and *Mn* at La Plata, where *Cd* concentration increased at a site closer to the river’s exit toward the sea, while the highest concentration of *Mn* was found at the midpoint of the river, having similar lower concentrations at the upper and lower reaches (Figure 3).

### Phthalates in superficial water samples

We found that of the six phthalates we tested for, selected as described above in the Materials and Methods section, only dibutyl phthalate (DBP) was found in concentrations that could be reliably detected by our GC-MS instrument. This particular phthalate was found only in samples from the La Plata River, at concentrations of 6, 8 and 3 μg/L (ppb), in the upper, midpoint, and lower reaches of the river, respectively.

## Discussion

The increase of the population and the development in Latin American countries and the Caribbean has caused a rapid urban growth, where many inhabitants have migrated and concentrated in urban zones. Puerto Rico is a densely-populated small tropical island where urban development along coastal metropolitan areas has consistently increased for the past 70 years, and where 40% of its land has been experiencing significant urban sprawl for the past 40 years [5,58–68]. As more people have moved from rural areas to urban zones, socio-demographic changes linked to a shift from agriculture to an industrial basis for the island’s economy, anthropogenic activities have become one of the main sources of pollution for nearby water systems. These activities, which include construction of housing, streets and highways, increased automobile traffic, use of septic tanks, and illegal dumping of waste from homes and businesses, have led to the emergence of contaminants such as esters, phthalates, derivatives of petroleum based fuel, and metals in many of the island’s varied ecosystems, including the rainforest, dry forest, beaches, cave systems, the karst, coast waters, lakes and rivers. Among these ecosystems, rivers are one of the most important, being a primary source of water considered safe for human consumption and use. Rivers are also the natural habitat of a wide variety of plants and animals, many of which are used as food or are part of the human food chain. Thus, resources from freshwater ecosystems directly contribute to the survival of the Puertorrican population and attract many people to settle on or near river banks. However, contamination of rivers due to increases in urbanization can in turn negatively impact the health of humans and other animal species.

Heavy metals and phthalates, as emergent pollutants, are of global concern due to the bioaccumulation, persistence and impact on ecosystems. It is known that inorganic compounds, including heavy metals, can have multiple toxic effects that negatively impact the function of the kidneys, lungs, heart, bones, skin, and various other target organs, including the nervous system [20]. There is also growing evidence that some phthalates, which can pass the blood brain barrier in mammals, may be linked with neural cellular and developmental deficits in rodents [69–71] and humans [72]. In Puerto Rico, where the incidence of premature births is amongst the highest in the world [73], results from longitudinal studies to monitor concentrations of contaminants found in urine among pregnant women indicate that certain urine phthalate metabolites are higher in Puerto Rico than those measured in women of reproductive age from the general US population [74]. Other reports link urinary phthalate metabolites in pregnant women in Puerto Rico with biomarkers of increased inflammation and oxidative stress [75]. Our research group has conducted experiments, as part of studies at the *Puerto Rico Center for Environmental Neuroscience*, where preliminary results indicate that short term exposure to low concentrations of phthalates may have an effect on dominant and submissive behaviors, as well as on general patterns of locomotive activity in freshwater crustaceans.

Regarding water pollution in rivers of Puerto Rico, the US Geological Survey, the agency in charge of monitoring water quality, has not issued reports on levels of specific contaminants such as heavy metals, plastics, or pharmaceutical products during the past 10 years. Very few studies on the impact of urbanism on contamination and the resulting degradation of river ecosystems have been conducted in tropical regions, particularly in the Caribbean [8,55]. Only last year was an initial study published on the impact of urbanism on water quality of Cuba’s Almendares River [76]. In Puerto Rico, the single environmental study conducted by a local investigator during the past decade [8] showed that the Río Piedras river has high concentrations of phosphates, potassium and magnesium, increments that were related with the increase in urbanization close to the river. However, little is known about other emergent pollutants, such as metals and phthalates, which are presently the focus of attention in Puerto Rico of various studies on potential effects on reproductive, developmental, and nervous system function in both humans and animals.

Our study focusing on the correlation between river contamination and urbanism shows that concentrations of heavy metals arsenic, barium, cadmium, manganese, and antimony are higher in urban rivers such as La Plata and the Río Piedras, compared to amounts found in a non-urban river, the Mameyes. The amounts of heavy metals obtained from superficial water samples were relatively low as compared with those reported by the US EPA as safe for drinking purposes, with only manganese being significantly (four times) higher than the allowed limit (US EPA: 5 μg/l).

In addition, dibutyl phthalate, a plasticizer and an emergent pollutant, was found at La Plata, but not the Río Piedras. Concentrations at La Plata ranged from 3 ppb at the river’s lower reach, to 6 ppb at the upper reach, and 8 ppb in the midpoint, the latter amount being above the EPA’s MCL for drinking water of 6 ppb. The difference between these two urban rivers is the presence of a dam in La Plata, located near the selected upper reach. The fact that the flow of the Río Piedras is not limited nor affected by any dam may contribute to a lower level of accumulation of certain contaminants, such as phthalates.

It is important to point out that this study was conducted during the rainy season of 2013; therefore, it is possible that high flow rates and disturbance in the river due to frequent rainfall could have led to re-suspension and downstream movement of pollutants into the water column. Thus, sampling of these rivers during the dry season will be very useful to compare outcomes and establish potential differences with the amounts of these emergent contaminants observed during the rainy season.

## Conclusion

Inductively Coupled Plasma and Gas Chromatography Mass Spectrometry were used to measure amounts of heavy metals and phthalates, respectively, in superficial water of three rivers of Puerto Rico: Mameyes (non-urban), Río Piedras (urban river without a dam), and La Plata (urban river with a dam). The urban rivers had significantly higher concentrations of heavy metals arsenic, barium, cadmium, manganese, and antimony, as compared with the reference non-urban river. Zinc at urban rivers was significantly lower than at the non-urban river. Manganese was the only metal found in concentrations higher than the maximum contaminant limits established for drinking water by the United States’ EPA. Of six phthalates amenable to measurement with the chosen protocol and instrumentation, only dibutyl phthalate was detected, only in the La Plata River, and at concentrations ranging from 3 to 8 parts-per-billion. These findings suggest that urbanism close to rivers of Puerto Rico is likely having an impact on water quality and thus further study to identify the potential sources, as well as the inclusion of these emergent contaminants on the list of chemicals regularly monitored by government agencies is justified.

We should be aware of the potential danger for these rivers of increasing concentrations of these emergent contaminants on both aquatic wildlife and humans experiencing prolonged or persistent exposure. Of particular concern is the high level of Mn observed in La Plata, since it is known that this metal can cross both the blood-brain barrier as well as the placenta during pregnancy, and at high concentrations can affect nervous system function and behavior [77]. Therefore, it is recommended that the relative high concentration of Mn in La Plata watershed be further monitored to assess its source, mobility, levels, bioavailability and persistence.

## Figures and Tables

**Table 1:** Most common phthalates available commercially.

*Dimethyl phthalate (DMP)
*Di(2-ethylhexyl) phthalate (DEHP)
*Dimethyl terephthalate (DMT)
*Dibutyl phthalate (DBP)
Di-n-octyl phthalate
*Butyl benzyl phthalate (BBP)
Diethyl phthalate
*Diisonyl phthalate (DnOP)
Mono-2-ethylhexyl phthalate
Mono-n-butyl phthalate
2-ethylhexanoic acid

**Table 2:** Physicochemical parameters of surface water samples obtained in a non-urban and two urban rivers on the northeast of Puerto Rico during the 2013 rainy season.

Parameters	Mameyes (NU)				Río Piedras (U)				La Plata (U)			
	Upper	Mid	Lower	Mean	Upper	Mid	Lower	Mean	Upper	Mid	Lower	Mean
pH	NM	7.60	7.70	7.65 ± 0.07	7.99	8.16	8.08	8.08 ± 0.09**	7.47	7.55	7.39	7.47 ± 0.08
Temperature	NM	25.5	26.7	26.1 ± 0.08	27.2	27.8	31.3	28.8 ± 2.2	29.4	30.1	27.8	29.1 ± 1.2
Dissolved Oxygen	NM	8.22	8.03	8.13 ± 0.13	7.36	7.92	9.15	8.14 ± 0.92	6.00	6.03	5.00	5.68 ± 0.59*

Temperature values of non-urban and urban rivers were not different statistically (p=0.1321). Alkaline pH at the Río Piedras river (U) was significantly different when compared with pH values at Mameyes (NU) and La Plata (U) (p=0.0007**). A multiple comparison test for dissolved oxygen resulted in significant reduction of DO in La Plata (U) when compared with DO measurements at the Mameyes (NU) and Río Piedras (U) rivers (p=0.0126). NU: non-urban river; U: urban river; NM: not measured; temperature is in °C, and dissolved oxygen is expressed as mg/L.

**Table 3:** Mean concentrations of heavy metals in surface water samples obtained from a non-urban and two urban rivers of Puerto Rico’s northeastern region, during the 2013 rainy season.

Metal	EPA (MCL) ppb	Mameyes(NU)			Río Piedras (U)			La Plata (U)		
		Upper Reach	Mid- Point	Lower Reach	Upper Reach	Mid- Point	Lower Reach	Upper Reach	Mid -Point	Lower Reach
Ag	* 100	NM	< 0.021	0.046 ± 0.044	< 0.021	< 0.021	< 0.021	< 0.021	< 0.021	< 0.021
As	10	NM	< 0.150	< 0.150	0.737 ± 0.063	0.563 ± 0.158	0.928 ± 0.024	1.177 ± 0.688	0.796 ± 0.130	1.717 ± 0.791
Ba	2,000	NM	45.433 ± 1.069	42.233 ± 0.764	107.667 ± 1.528	102.633 ± 3.564	105.333 ± 0.577	67.900 ± 2.138	60.667 ± 6.183	66.767 ± 1.079
Be	4	NM	< 0.027	0.056 ± 0.050	0.031 ± 0.007	< 0.027	< 0.027	< 0.027	< 0.027	< 0.027
Cd	5	NM	0.429 ± 0.033	0.409 ± 0.075	0.421 ± 0.049	0.503 ± 0.002	0.508 ± 0.025	0.233 ± 0.065	0.348 ± 0.026	0.439 ± 0.036
Cr	100	NM	1.843 ± 0.064	1.893 ± 0.076	1.927 ± 0.055	1.717 ± 0.071	1.663 ± 0.091	2.023 ± 0.546	1.747 ± 0.183	2.203 ± 0.099
Cu	1,300	NM	4.267 ± 0.356	3.640 ± 1.520	4.550 ± 0.537	3.970 ± 0.640	3.537 ± 0.522	3.950 ± 0.837	2.747 ± 0.283	5.100 ± 2.489
Mn	* 50	NM	5.387 ± 0.025	10.900 ± 0.436	43.267 ± 0.666	40.333 ± 1.102	32.400 ± 0.917	188.667 ± 4.509	207.333 ± 12.503	188.333 ± 5.508
Ni	**100	NM	3.183 ± 0.660	2.513 ± 1.300	2.567 ± 0.125	2.500 ± 0.092	2.643 ± 0.201	3.393 ± 1.171	1.993 ± 0.176	4.360 ± 1.948
Pb	15	NM	0.261 ± 0.103	0.213 ± 0.144	< 0.130	< 0.130	< 0.130	< 0.130	< 0.130	0.276 ± 0.253
Sb	6	NM	< 0.047	0.069 ± 0.038	0.121 ± 0.002	0.101± 0.002	0.110 ± 0.003	0.125 ± 0.025	0.088 ± 0.005	0.106 ± 0.012
Se	50	NM	0.393 ± 0.045	< 0.360	0.417 ± 0.010	0.425 ± 0.059	0.583 ± 0.057	1.740 ± 2.252	0.504 ± 0.193	3.210 ± 2.475
Tl	2	NM	< 0.020	< 0.020	< 0.020	< 0.020	< 0.020	< 0.020	< 0.020	< 0.020
Zn	*5,000	NM	9.733 ± 0.095	5.137 ± 2.662	< 3.600	< 3.600	< 3.600	4.773 ± 1.299	< 3.600	7.830 ± 3.948

Mean values ± standard deviation; MCL: Maximum Contaminant Level allowed in drinking water by the Environmental Protection Agency (EPA, 2009);

* Maximum secondary levels allowed in drinking water (EPA, 2009);

** Determined by State of California; NU: non-urban river; U: urban river; ppb: parts per billion; NM: no measured.

**Table 4:** Mean concentrations of heavy metals expressed as % of Maximum Contaminant Levels (MCL) allowed in drinking water in surface water samples obtained from a non-urban and two urban rivers of Puerto Rico’s northeastern region, during the 2013 rainy season.

Metal	EPA (MCL) ppb	Mameyes(NU)			Río Piedras (U)			La Plata (U)		
		Upper Reach	Mid- Point	Lower Reach	Upper Reach	Mid- Point	Lower Reach	Upper Reach	Mid -Point	Lower Reach
Ag	* 100	NM	0	0	0	0	0	0	0	0
As	10	NM	2	2	7	6	9	12	8	17
Ba	2,000	NM	2	2	5	5	5	3	3	3
Be	4	NM	1	1	1	1	1	1	1	1
Cd	5	NM	9	8	8	10	10	5	7	9
Cr	100	NM	2	2	2	2	2	2	2	2
Cu	1,300	NM	0	0	0	0	0	0	0	0
Mn	*50	NM	11	22	87	81	65	377	415	377
Ni	**100	NM	6	5	5	5	5	7	4	9
Pb	15	NM	2	1	1	1	1	1	1	2
Sb	6	NM	1	1	2	2	2	2	1	2
Se	50	NM	1	1	1	1	1	3	1	6
Tl	2	NM	10	10	10	10	10	10	10	10
Zn	*5,000	NM	0	0	0	0	0	0	0	0

% of MCL. MCL: Maximum Contaminant Level allowed in drinking water by the Environmental Protection Agency (EPA, 2009).

* Maximum secondary levels allowed in drinking water (EPA);

** Determined by State of California; NU: non-urban river; U: urban river; ppb: parts per billion; NM: no measured
